# Evolutionary Divergence in the Catalytic Activity of the CAM-1, ROR1 and ROR2 Kinase Domains

**DOI:** 10.1371/journal.pone.0102695

**Published:** 2014-07-16

**Authors:** Travis W. Bainbridge, Venita I. DeAlmeida, Anita Izrael-Tomasevic, Cécile Chalouni, Borlan Pan, Joshua Goldsmith, Alia P. Schoen, Gabriel A. Quiñones, Ryan Kelly, Jennie R. Lill, Wendy Sandoval, Mike Costa, Paul Polakis, David Arnott, Bonnee Rubinfeld, James A. Ernst

**Affiliations:** 1 Department of Protein Chemistry, Genentech, Inc., South San Francisco, California, United States of America; 2 Department of Cancer Targets, Genentech, Inc., South San Francisco, California, United States of America; 3 Center for Advanced Light Microscopy, Genentech, Inc., South San Francisco, California, United States of America; 4 Department of Structural Biology, Genentech, Inc., South San Francisco, California, United States of America; University of Georgia, United States of America

## Abstract

Receptor tyrosine kinase-like orphan receptors (ROR) 1 and 2 are atypical members of the receptor tyrosine kinase (RTK) family and have been associated with several human diseases. The vertebrate RORs contain an ATP binding domain that deviates from the consensus amino acid sequence, although the impact of this deviation on catalytic activity is not known and the kinase function of these receptors remains controversial. Recently, ROR2 was shown to signal through a Wnt responsive, β-catenin independent pathway and suppress a canonical Wnt/β-catenin signal. In this work we demonstrate that both ROR1 and ROR2 kinase domains are catalytically deficient while CAM-1, the *C*. *elegans* homolog of ROR, has an active tyrosine kinase domain, suggesting a divergence in the signaling processes of the ROR family during evolution. In addition, we show that substitution of the non-consensus residues from ROR1 or ROR2 into CAM-1 and MuSK markedly reduce kinase activity, while restoration of the consensus residues in ROR does not restore robust kinase function. We further demonstrate that the membrane-bound extracellular domain alone of either ROR1 or ROR2 is sufficient for suppression of canonical Wnt3a signaling, and that this domain can also enhance Wnt5a suppression of Wnt3a signaling. Based on these data, we conclude that human ROR1 and ROR2 are RTK-like pseudokinases.

## Introduction

Receptor tyrosine kinase-like orphan receptors (ROR) 1 and 2 are among the most widely studied non-canonical Wnt receptors of the receptor tyrosine kinase (RTK) family and ROR genes are conserved in animals from *C. elegans* to humans [1]. Mutations that affect localization and activity of ROR2 cause the developmental defects Robinow syndrome and brachydactyly type B [2]. ROR2 has been linked to a number of human cancers and is thought to increase cellular migration though increased expression [3], [4]. Further, expression of ROR1 is highly upregulated in chronic lymphocytic leukemia (CLL) [5]–[7], acute lymphoblastic leukemia (ALL) [8] and mantle cell lymphoma (MCL) [9]. Deletion of either ROR1 or ROR2 in mice is lethal, resulting in skeletal, pulmonary and cardiac developmental defects [10].

**Figure 1:**
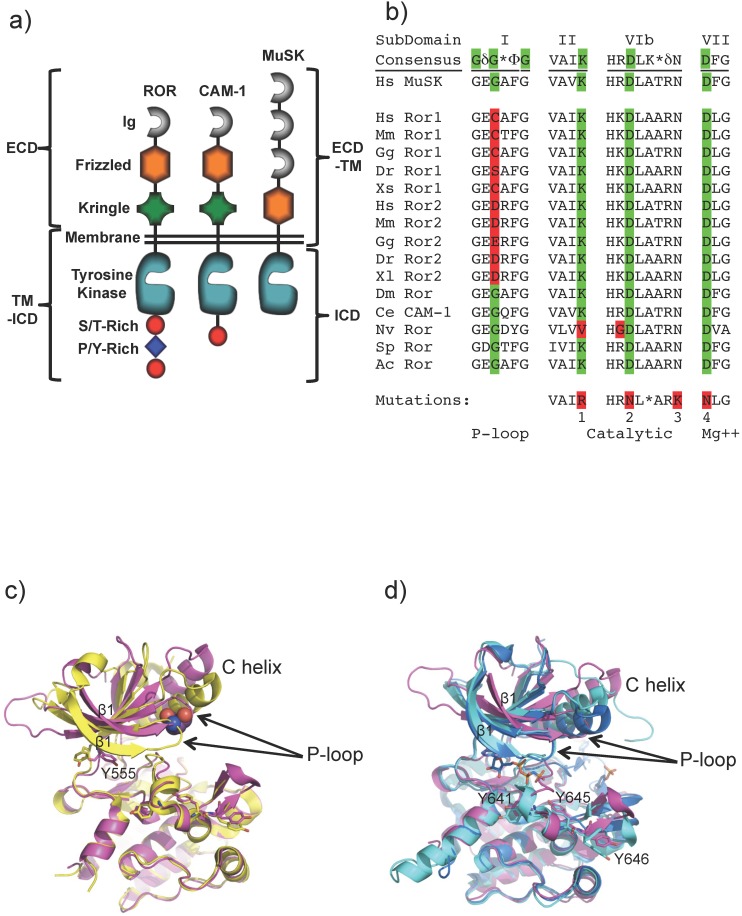
Domain structures of ROR and MuSK receptors, kinase mutations in ROR and structural analysis of the ROR2 kinase domain. a) Domain structures of the ROR, CAM-1 and MuSK receptors showing boundaries of constructs used in experiments. b) Alignment of consensus sequence for regions of kinase domain critical for enzyme activity. Variance from consensus kinase sequence found in ROR is highlighted red; consensus residues are highlighted green. Hs; *Homo sapiens*, Mm; *Mus musculus*, Gg; *Gallus gallus*, Dr: *Danio rerio*, Xl; *Xenopus laevis*, Xs; *Xenopus* (Silurana) *tropicalis*, Dm; *Drosophila melanogaster*, Ce; *Caenorhabditis elegans*, Ac; *Aplysia californica* (sea slug), Nv; *Nematostella vectensis* (sea anemone), Sp; *Strongylocentrotus purpuratus* (sea urchin). c) Alignment of human ROR2 kinase domain (PDB ID: 3ZZW pink) with rat MuSK kinase domain (PDB ID: 1LUF yellow, RMSD C-terminal lobe only, 0.4Å, all atom 0.73 Å). ROR2 Tyrosine 555 is shown in the canonical adenine binding site in the apo structure. d) Alignment of human ROR2 kinase domain (pink) with apo IGF-R (PDB ID: 1P4O cyan) and phosphorylated, activated IGF-R (PDB ID: 1K3A, sky blue, RMSD to ROR2 C-terminal lobe only, 0.8Å, all atom 1.5Å). Tyrosines in the ROR2 activation loop are numbered. The non-hydrolyzable ATP analogue AMP-PCP is shown in dark blue. Human ROR2 kinase domain and human IGF-R kinase domain share approximately 40% sequence identity.

The RORs share significant domain similarity to Muscle Specific Kinase (MuSK) receptor (Figure 1a), which is activated by the extracellular matrix protein Agrin and the co-receptor LRP4, causing cytoskeleton rearrangement and formation of myotubes [11]. MuSK activity plays a role in neuromuscular junction formation and neural crest cell migration through processes thought to be regulated by Wnt signaling [12].

Wnt signaling is mediated through several pathways: a canonical β-catenin dependent pathway, and multiple non-canonical β-catenin independent pathways which remain poorly understood [13], [14]. The canonical signaling pathway is initiated by binding of Wnt ligands to the Frizzled (FZD) and low-density lipoprotein receptor-related protein (LRP) co-receptors and results in β-catenin stabilization, nuclear translocation of β-catenin and promotion of transcription through the lymphoid enhancer-binding factor/T cell factor (Lef/Tcf) coactivators. Canonical Wnt signaling plays an essential role in embryonic development and the maintenance of adult stem cells in regenerative tissue compartments. Activated canonical Wnt signaling resulting from mutations in genes encoding β-catenin or adenomatous polyposis coli (APC) that disrupt β-catenin degradation has been observed in many types of cancer [15].

Non-canonical Wnt pathways can be stimulated by the ligand Wnt5a, however, signaling proceeds independently of β-catenin, through several alternate downstream effectors, many of which have been linked to the generation and maintenance of planar cell polarity (PCP) (7). ROR1 and ROR2, which share approximately 50% sequence identity [1], have a cysteine-rich domain (CRD) similar to that in FZD receptors [16], [17] and bind Wnt5a ligand [18]. Wnt5a has been shown to establish PCP by stimulating Vangl2 phosphorylation through ROR2 [19]. Wnt5a has also been shown to stimulate association and internalization of FZD2 or FZD7 with ROR2 and activate the small G-protein RAC1 following receptor endocytosis [4], [20]. Additionally, Wnt5a and ROR2 can regulate phosphorylation of transcription factors by effectors such as JNK [21]. In most of these examples, non-canonical Wnt signaling pathways oppose the canonical β-catenin Wnt signaling pathway, although the mechanisms remain controversial.

The kinase domains of the vertebrate ROR proteins contain non-consensus cysteine, aspartic or glutamic acid residues in the glycine rich P-loop that may hinder ATP binding and disrupt enzymatic activity (Figure 1b) [22]. Likewise, the ROR homolog from at least one other species, *Nematostella vectensis* (sea anemone), is predicted to be inactive based on substitution of a valine residue for the catalytic lysine in the VAIK ATP binding motif. In contrast, the closely related human receptor, MuSK, or ROR proteins in lower organisms, like the *C. elegans* CAM-1 [23] and the *D. melanogaster* ROR retain consensus kinase motifs. The kinase domains of these receptors share 40 to 45% sequence identity with ROR.

The catalytic effects of substitutions of larger amino acids in the second glycine of the kinase P-loop are incompletely explained by the available structural data. Structural alignments of the C-terminal lobes of the kinase domain of ROR2 and the ATP-free, apo form of MuSK [24], are closely related with an RMSD of less than 1 Å for all atoms (Figure 1c). In both structures, the catalytic lysine (Lys507 in ROR2) and the magnesium-coordinating aspartic acid from the DFG region (Asp633 in ROR2) are in the inactive conformation, as is often observed in kinase structures solved in the apo state. The ROR2 Tyr555 side-chain in the hinge region also partially occludes the adenine-binding site. In contrast, the equivalent tyrosine residue in MuSK is oriented away from the active site and would not block adenine/ATP binding. In addition, segments of the N-terminal lobe including the P-loop and sheet β1 of ROR2 are in a relatively open conformation creating a larger ATP binding site with fewer stabilizing interactions relative to those available in MuSK [24] and other kinases; this open conformation of ROR2 may result from the aspartic acid substitution in the P-loop. A comparison of ROR2 kinase domain to the related kinase domain of insulin-like growth factor receptor [24] (IGF-R) in the apo [25] and phosphorylated, ATP-bound state [26] is shown in Figure 1d. In these structures, the P-loop and sheet β1 of the IGF-R N-terminal lobe are in almost identical locations in both the ATP-bound and ATP-free structures and thus are positioned to pre-form part of the ATP binding site, demonstrating that a conformational rearmament of the P-loop is not required for ATP binding. However, kinase domains are highly flexible and there is no obvious structural rationale that would prevent ROR1 or ROR2 from undergoing a structural rearrangement to accommodate the non-consensus P-loop residues.

An accurate understanding of the function and enzymatic activity of the ROR kinase domain is essential to defining its role in disease. We therefore evaluated the enzymatic activity of isolated ROR1 and ROR2 kinase domains which have been overexpressed in insect cells and purified to greater then 95% homogeneity. Previous studies with crudely purified, immunoprecipitated ROR1 that may contain co-factors or associated kinases have produced conflicting results, with one study demonstrating the presence of ROR1 kinase activity [1] and another refuting this result [27]. With respect to ROR2, studies have shown the presence of phosphorylation in response to Wnt5a stimulation [28]–[30], but these studies did not demonstrate ROR2 autophosphorylation, nor were any tyrosine phosphorylation sites mapped within the activation loop.

In the current work, *in vitro* biochemical activity of the highly purified cytoplasmic domains of ROR1 and ROR2, or the positive control CAM-1 and MuSK indicate extremely weak activity for either human ROR kinase domain; comparable to that of classic kinase-inactivating mutants of MuSK and CAM-1. In fact, this lack of robust kinase activity of human ROR is characteristic of a pseudokinase, as suggested from the analysis of the protein sequence, unlike CAM-1 and MuSK. Further, we demonstrate using quantitative mass spectrometry that Wnt5a activation of ROR1 is not associated with kinase activation of the receptors in mammalian cells. Full-length and truncation constructs of ROR1 and ROR2 were evaluated in a cell-based assay for Wnt reporter activity under identical conditions to the quantitative mass spectrometry experiments, demonstrating that enhanced inhibition of canonical Wnt signaling by either ROR does not require the presence of the kinase domain. These biochemical data, combined with the phylogenetic data, suggest a divergence of selection pressure on mammalian ROR during evolution to retain the robust phosphotransferase activity of CAM-1 and MuSK, and point to the dominant function of the ROR kinase domain as a pseudokinase scaffold for recruiting other proteins active in cell polarity processes.

## Experimental Procedures

### Protein Isolation and Analysis

Wnt3a and Wnt5a protein used for *in vitro* assays was purified from L cells transfected with mouse Wnt3a (American Type Culture Collection) as previously described [31] or purchased from R&D systems (Minneapolis, MN).

A recombinant baculovirus/Sf9 cell system was used to express the wild type and mutant intracellular domains (ICD) of ROR1, ROR2, MuSK or CAM-1, encoding residues C428-L937, C428-A943, R519-V869 or R491-D928 respectively (Figure 1a), with an N-terminal six-histidine tag and an additional N-terminal GST tag for MuSK. For protein extraction, cells were homogenized in lysis buffer (50 mM Tris pH 8.25, 400 mM NaCl, 10 mM imidazole, 20% glycerol, 2 mM Tris(2-carboxyethyl)phosphine, 0.2 mM sodium orthovanadate, 5 mM MgCl_2_, 5 units benzonase/mL, Roche complete protease inhibitor cocktail without EDTA) and disrupted by nitrogen decompression. After ultracentrifugation of the lysate, the supernatant was filtered and then purified by nickel-affinity chromatography followed by size-exclusion chromatography. All lysis and purification steps were performed on ice or at 4°C. Protein identities were confirmed by mass spectrometry.

### Autophosphorylation and Kinetics of Activity

For detection of autophosphorylation, the wild type and mutant ICDs of receptors ROR1, ROR2, CAM-1 and MuSK were incubated overnight, approximately 18 hours, at 4°C, with 2 mM ATP, 20 mM MgCl_2_, and 0.2 mM sodium orthovanadate. The controls were incubated likewise, without ATP. The proteins were then analyzed by immunoblotting.

Steady-state kinetic measurements were carried out by phosphocellulose paper binding assay [32] using 10 µM unphosphorylated or phosphorylated enzyme. For determination of the *K_m(peptide)_* 0–5 mM activation loop peptide (CAM-1, RTSYGSDYYKK; ROR1, SREIYSADYYRR; IRK, TRDIYETDYYRK), 5 mM ATP and 20 mM MgCl_2_ were used. For determination of *K_m(ATP)_*, 0–10 mM ATP and 20 mM MgCl_2_ or 0–5 mM ATP and 5 mM MnCl_2_ were used, both in the presence of 5 mM peptide. All reactions were carried out at 20°C and included [γ−^32^P]ATP. Kinetic parameters were determined by fitting the initial rate data (<10% total substrate turnover) to the Michaelis-Menten equation using GraphPad Prism software. Phosphorylated CAM-1 was generated by pre-incubating purified enzyme with 2 mM ATP, 20 mM MgCl_2_, and 0.2 mM sodium orthovanadate overnight at 4**°**C. Phosphorylation was confirmed by anti-phosphotyrosine immunoblot and intact mass spectrometry.

### Protein Analysis by Immunoblot

For detection of tyrosine phosphorylation, the proteins were resolved on 4–20% SDS-polyacrylamide gels and transferred to nitrocellulose membranes. The membranes were probed with anti-phosphotyrosine primary antibody (Millipore, 4G10 Platinum-HRP), followed by IRDye 800CW Rabbit anti-HRP secondary antibody (LI-COR). The blots were imaged and quantitated using an Odyssey Infrared Imaging System (LI-COR) and Image Studio software.

Expression of receptor constructs in cells used for the luciferase assay was determined by immunoblot detection of receptor proteins using anti-Flag M2 antibody (Sigma) and 10 µl of the lysate from the luciferase assays. Cytosolic β-catenin, in cells transfected with empty vector, ROR1, ROR2 or MuSK constructs and treated with Wnt3a and/or Wnt5a was detected using the anti-β-catenin monoclonal antibody (BD Biosciences). For this assay, cells were lysed in a hypotonic buffer (20 mM Tris HCl pH 8.0, 1 mM EDTA, 50 mM NaF, 1 mM Na_3_VO_4_, 1 mM DTT with protease inhibitors) and the cytosolic fraction used for electrophoresis. The proteins were electrophoresed on 4–20% TGX gels (Bio-Rad), followed by transfer to PVDF membrane. Blots were incubated with the primary antibodies (overnight), and anti-mouse or rabbit IR conjugated secondary antibodies (1 hour, Invitrogen) and the proteins visualized and quantitated using the Odyssey Infrared Imaging System (LI-COR).

### Phosphorylation Site Mapping

Coomassie Blue stained SDS-PAGE gel bands corresponding to ROR1, ROR2 and CAM-1 ICDs were excised, reduced, cysteine-alkylated, and digested with trypsin *in situ* [33]. Aliquots of the resulting peptides were enriched for phosphopeptides using TiO_2_ affinity chromatography (Titansphere; GL Sciences Inc., Torrance, CA). Peptides before and after phospho-enrichment were analyzed by capillary reverse phase liquid chromatography tandem mass spectrometry on an orbitrap mass spectrometer (Orbitrap XL; Thermo Fisher, San Jose, CA). Mass spectra were recorded in data-dependent experiments whereby eight collision-induced dissociation product ion spectra were collected per full MS scan [34]. Mass spectra were searched against a database of human proteins using the Mascot program (Matrix Science) and putative phosphorylation sites manually verified.

### Phosphopeptide Quantitation

The ROR1 tryptic peptide (EIYSADYYR) was quantified in its unphosphorylated form and as potentially singly phosphorylated on each of its three tyrosines by stable isotopes dilution mass spectrometry. Synthetic peptides labeled with stable isotope that gave each form of the peptide a distinct mass were procured (Cell Signaling Technologies; Danvers, MA). These were added to ROR1 tryptic digests to yield 133 fmol per duplicate LC-MS injection, performed as above. Peak areas were extracted from the Orbitrap full MS scans and endogenous peptide amounts were calculated relative to the synthetic peptide standards. Where no peak corresponding to the endogenous phosphopeptide was observed, an upper bound for its concentration was calculated based on the values of background peaks of the same mass to charge ratio.

### Expression Constructs, Mammalian Cell Culture, Transfection, and Luciferase Assays

The ROR1 FL (Q30-L937), ROR1 ECD (Q30-E403), ROR1 ECD-TM (Q30-C428), ROR1 TM-ICD (D392-L937), ROR2 FL (E34-A943), ROR2 ECD (E34-G403), ROR2 ECD-TM (E34-C428), ROR2 TM-ICD (S395-A943) and MuSK FL (E22-V783 of isoform 2) (Figure 1a) constructs were generated by PCR of their respective cDNAs and subsequent subcloning into a pRK-5 expression vector modified to encode the signal sequence of HSV glycoprotein D, followed by an amino-terminal FLAG tag.

Human kidney epithelial 293 (HEK293) cells were grown in 50∶50 high-glucose DMEM and Ham’s F12 with 10% fetal bovine serum (FBS). For luciferase assays, 0.6×10^4^ HEK293 cells were plated per well of a 24-well dish (Nunc) 24 h before transfections. Cells were transfected with 0.125 mg of the indicated receptor construct and 0.125 mg of TB23 mix with Fugene 6 (Roche) as described [35]. After 24 h, the medium was changed and cells were left untreated or treated with purified Wnt3a or Wnt5a for 20–24 hr. Cells were harvested in 60 µl of lysis buffer (20 mM Tris at pH 8.0, 137 mM NaCl, 1 mM EGTA, 1% Triton X-100, 10% glycerol, 1.5 mM MgCl_2_, 1 mM DTT, 50 mM NaF, 1 mM Na_3_VO_4_ and protease inhibitor cocktail) and 10 µl samples were assayed in duplicate using the Dual-Glo Luciferase Assay kit (Promega) and detected in an Envision (Perkin-Elmer). Firefly luciferase activity was normalized against Renilla luciferase activity and the data normalized to this ratio obtained in untreated cells.

## Results

### Purified ROR, but not CAM-1 and MuSK, lack robust kinase activity *in vitro*

To gain greater biochemical insight into the functions of the ROR kinase domains and resolve the controversy around the kinase activity, we expressed and isolated wild type and mutant forms of the full intracellular domains (ICDs) from human ROR1, ROR2, MuSK and *C. elegans* CAM-1 (Figure 1a). Further, we generated single point mutations in ROR kinase domains to restore the canonical GxGxxG, P-loop, and mutations in MuSK and CAM-1 to substitute the non-consensus P-loop residues of ROR1 and ROR2. Finally, common kinase-inactivating mutations of MuSK and CAM-1 were generated as negative controls (Figure 1b). All purified proteins are greater than 95% pure as characterized by Coomassie stained gel (Figure S1). MuSK has been previously shown to be an active tyrosine kinase [30] while the *in vitro* kinase activity of CAM-1 has not previously been reported.

**Figure 2:**
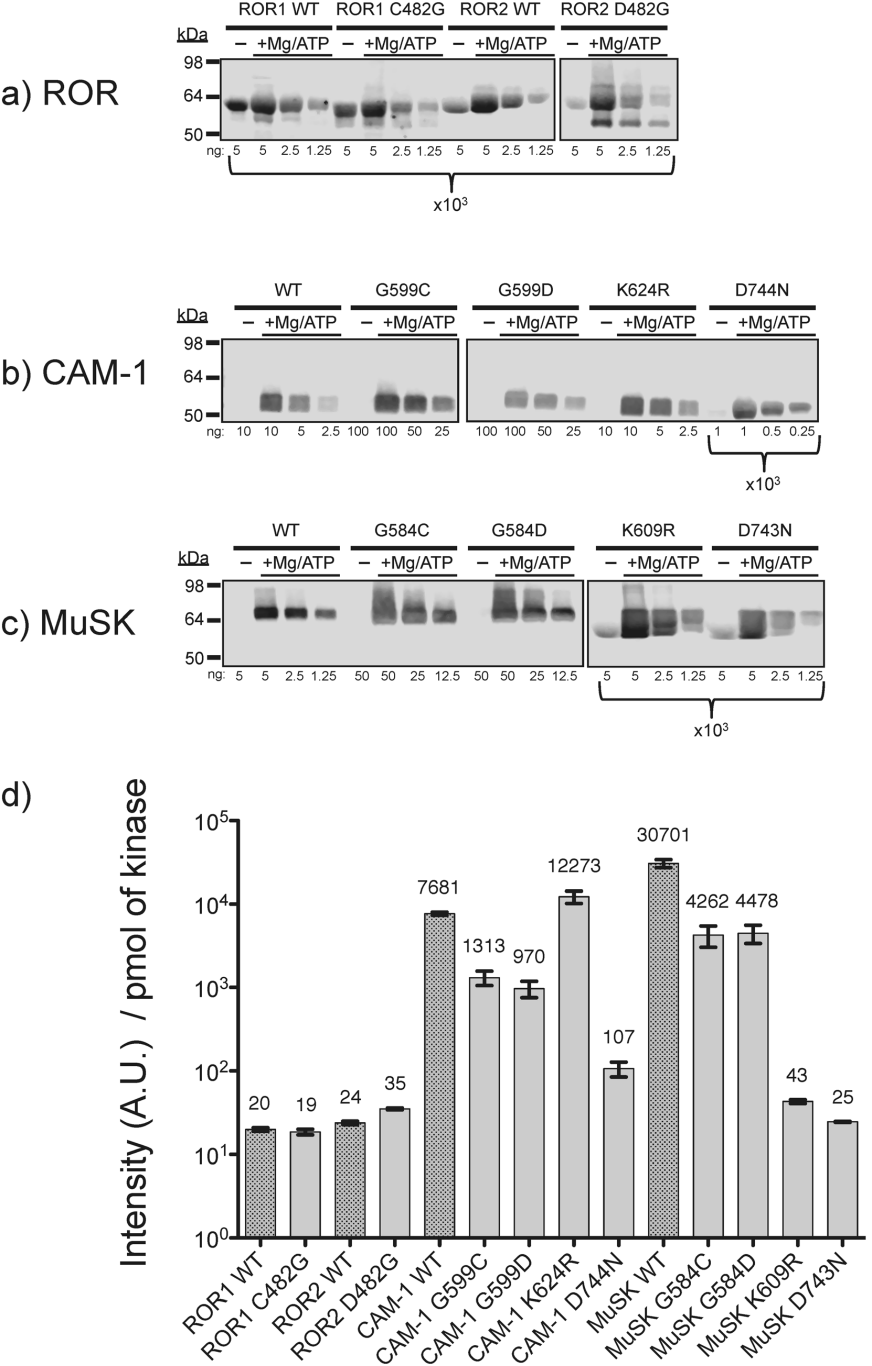
ROR shows limited residual tyrosine autophosphorylation *in vitro.* a) ROR, b) CAM-1, c) MuSK and d) Quantitation of signal intensity per pmol of kinase from the above immunoblots. Error bars represent standard error of the mean. ROR proteins were expressed in insect cells and isolated to >95% purity. Phosphotyrosine was detected by anti-phosphotyrosine, after incubation in the presence or absence of magnesium and ATP. The amount of protein loaded is indicated below each lane in nanograms. These data are representative of two or more independent protein expression and isolation experiments. Quantitation data of experimental replicates can be found in Figure S2.

After overnight incubation with magnesium and ATP, the wild type kinase domains of ROR1 and ROR2 show 100 and 1000 fold less tyrosine phosphorylation than CAM-1 and MuSK, respectively (Figures 2 & S2). Restoration of the consensus P-loop glycine in the ROR1/2 C/D482G mutants causes a reproducible 1.5 to 3-fold improvement in the catalytic activity of ROR2 but no consistent effect of the activity of ROR1. However, CAM-1 and MuSK mutants harboring the non-consensus ROR P-loop residues lose more than 85% autophosphorylation kinase activity with respect to wild type. The negative controls, a catalytic lysine mutant of MuSK (K609R) and the magnesium-binding mutants (CAM-1 D744N & MuSK D743N) were less active than the P-loop mutants, but retained a detectable level of tyrosine phosphorylation comparable to wild type and mutant ROR. Interestingly, the catalytic lysine mutant in CAM-1 (K624R) had little to no average change in activity. However, CAM-1 contains two adjacent, potentially catalytic lysine residues in Subdomain II and it is likely that K625 is compensating for the mutation K624.

To further characterize the catalytic activity of ROR1 and ROR2, we determined *in vitro* steady state kinetic parameters for peptide phosphorylation activity using [γ−^32^P]ATP in a phosphocellulose paper binding assay. Peptides from the activation loop of CAM-1, ROR1 or insulin receptor kinase (IRK) were evaluated as substrates. CAM-1 was able to phosphorylate all three peptides. In contrast, neither ROR1 nor ROR2 demonstrated any phosphorylation activity in identical conditions (Table 1). We were, however, able to determine the kinetic constants for both wild type and K624R CAM-1 in the presence of either magnesium or manganese in both the phosphorylated and unphosphorylated states. Wild type CAM-1 *K_m(ATP)_* in the presence of magnesium is, within experimental error, identical to that previously reported for unphosphorylated MuSK and the *K_m(ATP)_* is further reduced in the presence of manganese, as has been observed for other tyrosine kinases [36], [37]. CAM-1 demonstrated no additional activation upon phosphorylation, which contrasts with the ∼100 fold improvement observed in MuSK [24] but is consistent with a high *K_m(peptide)_* and the null phenotype observed for the kinase dead version of CAM-1 [23].

**Table 1:** Comparison of kinetic parameters for MuSK, CAM-1, ROR1 and ROR2 tyrosine phosphorylation activity.

Kinase	*K_M(peptide)_*	*K_M(ATP)_*	*K_cat_*	*K_cat_*/*K_M(ATP)_*
	(mM)	(mM)	(min^−1^)	(min^−1^mM^−1^)
MuSK, Unphosphorylated^a^	1.58±0.24	3.40±0.27	0.47±0.03	0.14
MuSK, Phosphorylated^a^	0.68±0.07	0.38±0.12	108.0±0.1	284
CAM-1, Unphosphorylated, Mg^2+^	>5	3.53±0.34	2.63±0.11	0.75
CAM-1, Unphosphorylated, Mn^2+^	ND	0.45±0.09	2.46±0.16	5.47
CAM-1, Phosphorylated, Mg^2+^	>5	3.35±0.57	2.53±0.18	0.76
CAM-1, Phosphorylated, Mn^2+^	ND	0.63±0.07	2.64±0.10	4.19
CAM-1 K624R, Mg^2+^	ND	6.69±1.12	0.20±0.02	0.03
CAM-1 K624R, Mn^2+^	ND	0.74±0.18	0.51±0.04	0.69
CAM-1 D744N, Mg^2+^ or Mn^2+^	ND	>15	<0.0005	<0.00003
CAM-1 G599C, Mg^2+^ or Mn^2+^	ND	>15	<0.0005	<0.00003
CAM-1 G599D, Mg^2+^ or Mn^2+^	ND	>15	<0.0005	<0.00003
ROR1, Mg^2+^ or Mn^2+^	>5	>15	<0.0005	<0.00003
ROR1 C482G, Mg^2+^ or Mn^2+^	ND	>15	<0.0005	<0.00003
ROR2, Mg^2+^ or Mn^2+^	ND	>15	<0.0005	<0.00003
ROR2 D482G, Mg^2+^ or Mn^2+^	ND	>15	<0.0005	<0.00003

CAM-1 and ROR data were obtained from a [γ−^32^P]ATP-based phosphocellulose binding assay. Data are from three technical replicates, ± standard error. ND, Not determined. ^a^MuSK data are from [24].

In order to determine the specific locations of the phosphorylation detected by immunoblot analysis, the phosphosites of wild type ROR1, ROR2 and CAM-1 were mapped using electrospray mass spectrometry. Tyrosine phosphorylation within the respective activation loops and additional phosphorylation sites within the kinase and serine/threonine-rich domains identified from the eukaryotic expression source are shown in Table S1. No activation loop tyrosine phosphorylation of ROR1, ROR2 and CAM-1 ICDs was detectable prior to incubation with ATP and magnesium. However, the observed phosphorylation sites were detected only after chromatographic enrichment, suggesting that phosphorylation in these samples is an uncommon event potentially indistinguishable from background. We therefore sought to evaluate the phosphorylation of ROR1 in a cellular context using the phosphotyrosine site determined *in vitro*.

### Intracellular ROR1 lacks phosphorylation activity

**Figure 3:**
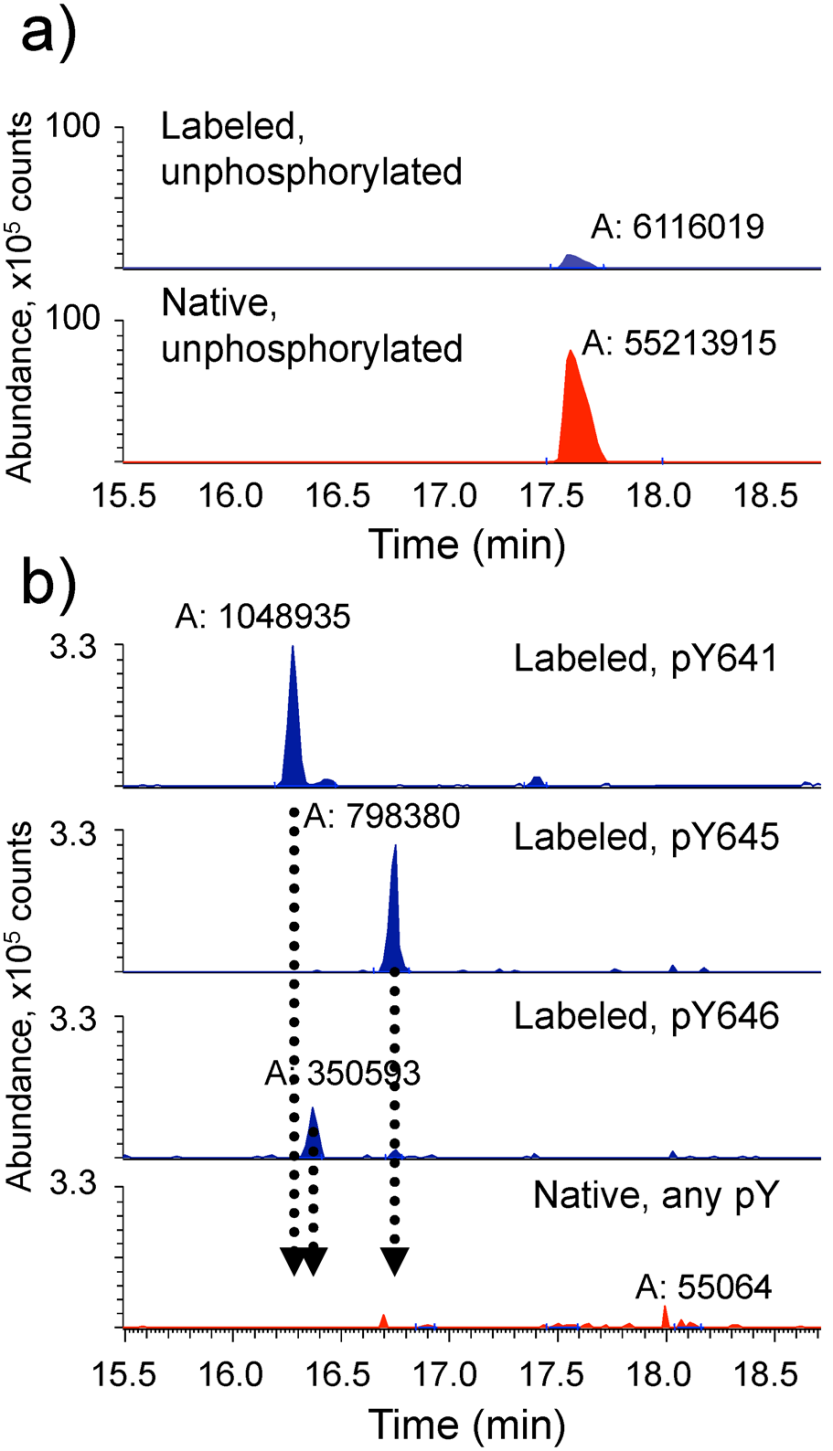
ROR1 activation loop is not phosphorylated upon Wnt5a stimulation of cells. Quantitative mass spectrometry reveals little or no phosphorylation of the ROR1 activation loop following Wnt5a treatment. Stable isotope labeled synthetic peptides corresponding to the kinase activation loop of ROR1 were used to determine the extent of phosphorylation of ROR1 following Wnt5a treatment of 293 cells. a) Representative chromatograms of isotopically labeled apo-peptide (Blue) and native isolated apo-peptide (Red). b) Representative chromatograms of isotopically labeled phosphopeptides (Blue) and endogenous isolated peptides for each of the three tyrosines in the activation loop (Red). A, Integrated peak area. Dotted lines indicate expected peak times in the native chromatogram. Y-axis scale is reduced in b) to illustrate the limits of detection and quantitation.

To evaluate if Wnt5a could activate the kinase domain of ROR1 expressed in HEK293 cells, we transfected full-length FLAG tagged ROR1 and treated cells with 100 µg/ml of Wnt5a. Isotopically labeled synthetic peptides (described in Methods) identified from the phospho-mapping of the ROR1 catalytic domain, including singly phosphorylated fragments from the kinase domain activation loop, were then used to determine the extent of tyrosine phosphorylation in the activation loop of ROR1. Following anti-FLAG immunoprecipitation, phosphorylation of tyrosine residues 641, 645 and 646 of the activation loop of ROR1 was not detected above a limit of 1 part per 100 (Figure 3, Table 2). We then extended our analysis of the ROR1 intracellular domain to identify any additional residues that might be phosphorylated following treatment with Wnt5a. No tyrosine phosphorylation was identified, even following targeted analysis for tyrosine phosphorylation.

**Table 2:** Quantitation of cellular ROR1 phosphorylation.

Total unphosphorylated	4566 fmol	±48 fmol
Total pY641	<16	±2.5
Total pY645	<19	±4.6
Total pY646	<40	±2.2
**Total peptide**	**<4641**	**±24**
pY641 is less than	0.34%	of total peptide
pY645 is less than	0.40%	of total peptide
pY646 is less than	0.86%	of total peptide

Data from three technical replicate peptide samples from the ROR1 activation loop are analyzed to determine the quantitation limit for phosphotyrosine autophosphorylation of ROR1 overexpressed in and isolated from Wnt5a-treated HEK293 cells.

### ROR kinase activity is not required for inhibition of canonical Wnt signaling

**Figure 4:**
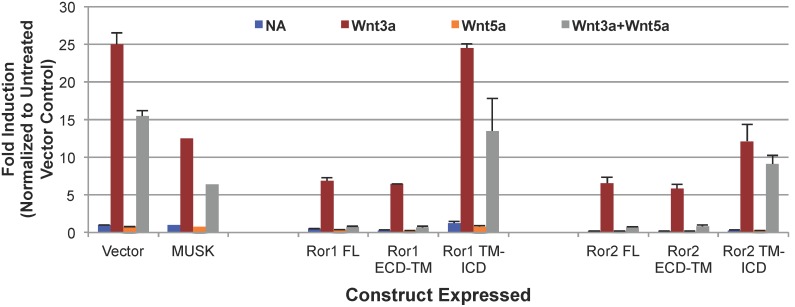
ROR ICD is not required for suppression of canonical Wnt signaling. ROR1 and ROR2 inhibit Wnt3a-stimulated transcription of a luciferase reporter from a Lef/Tcf promoter and this activity is enhanced by Wnt5a ligand. The ROR1 or ROR2 ECD alone can suppress canonical Wnt signaling when anchored to the plasma membrane. The luciferase reporter data presented in this figure are representative of at least three independent experiments and are normalized to untreated vector transfected cells. Error bars represent standard error.

ROR1 and ROR2 are known to act as Wnt5a receptors [29], [38], however, the function of the kinase domains of these receptors in intracellular signaling is unclear. Both ROR1 and ROR2 alone inhibit Wnt3a stimulation of TOPbrite Wnt reporter activity in HEK293 cells (Figure 4), consistent with previous reports [38]. Addition of Wnt5a modestly inhibits Wnt3a stimulated TOPbrite activity in control cells, and this effect is greatly enhanced by expression of either ROR1 or ROR2, as compared to the related MuSK receptor used as control (Figure 4) and other unrelated control receptors (data not shown), suggesting that this repressive effect is specific to ROR. To identify the domains of ROR that are involved in inhibiting Wnt3a activity, we generated truncated receptor constructs containing the extra-cellular domain (ECD) or the ICD, tethered to the plasma membrane by the native transmembrane domain (TM) (Figure 1a). Cell surface expression of these constructs was confirmed by FACS analysis, using an anti-FLAG antibody (Figure S3a), while receptor protein expression in luciferase assay samples was determined by immunoblotting (Figure S3b). Expression of the ECD-TM constructs of ROR1 and ROR2 results in inhibition of the canonical Wnt3a signal in HEK293 cells (Figure 4) in the absence of Wnt5a. Antagonism of canonical Wnt3a signaling by the ROR ECD-TM, but not the TM-ICD, is further enhanced by the addition of Wnt5a ligand. In fact, both full length ROR and ROR ECD-TM completely block Wnt3a signaling when stimulated with Wnt5a; an effect not seen in our controls.

## Discussion

A number of molecular events have been proposed to explain ROR signaling mechanisms, including tyrosine phosphorylation by the ROR kinase domain [29], complex formation and phosphorylation of Vangl2 [19], complex formation and internalization with FZD receptors [39], [40] and Dsh oligomerization [40]. Given the overall sequence identity between ROR and the conventional RTKs CAM-1 and MuSK, which are also involved in cytoskeletal rearrangements, and the observation that ROR2 and Wnt5a stimulate Dsh phosphorylation [41], it might be assumed that Wnt5a and ROR should launch a phosphorylation cascade. Our data demonstrate that if such a cascade exists in vertebrates, it is unlikely to require enzymatic tyrosine phosphorylation by the ROR kinase domains, consistent with one earlier report [40]. In fact, CAM-1 kinase activity is not necessary to maintain cell polarity, cell migration and asymmetric cell division in *C. elegans* [23], [42]. Additionally, in mammalian cells, ROR Kinase activity is not required for stimulation of filopodia formation, cytoskeletal reorganization and cell migration in response to Wnt5a [43]. Further, it is clear from our Wnt reporter assay data that the kinase domain of ROR is not essential for its role in suppression of canonical Wnt activity. *In vitro,* the efficiency of ROR1 and ROR2 tyrosine phosphorylation catalysis, as determined by anti-phosphotyrosine immunoblot is 100 to 1000 fold less than that observed for the *C. elegans* ROR homolog CAM-1 or human MuSK. In addition, enzyme kinetics experiments for peptide tyrosine phosphorylation by the kinase domains ROR1 and ROR2 demonstrated no activity within the quantification limits of the assay (K_M(ATP)_>15 mM), in contrast to CAM-1 and MuSK (33). While these data alone are not conclusive evidence of a complete lack of catalytic potential, the biological significance of such a high K_M(ATP)_ at typical cellular ATP concentrations is dubious, at least on common receptor tyrosine kinase substrates. Previous reports have associated kinase activity with ROR that has been immunoprecipitated from crude cell extracts, a condition where cofactors or associated kinases might also co-precipitate [1], [29]. However, using highly purified material prepared in a manner similar to ours, a recent study has shown the ROR1 kinase domain to have no detectable Mg^++^-binding nor ATP-binding activity in a thermal-shift assay [44], consistent with our data. Wnt5a-dependent *trans*-phosphorylation of ROR by kinases has been noted by researchers [28] and may explain the previously observed ROR cellular tyrosine phosphorylation.

The kinetics and autophosphorylation experiments in the current report were performed with isolated protein of greater than 95% purity. It is possible that, in contrast to CAM-1, human ROR kinase activity may require a trans-activating cellular cofactor or that an autoregulatory domain inhibits activity of the constructs used in our biochemical analysis, as has been shown for other RTKs [45]. However, Wnt5a-induced activation of ROR1 tyrosine kinase activity can be ruled out by our quantitative mass spectrometry experiments using full length ROR1 expressed in HEK293 cells and activated with Wnt5a. Under these conditions less than 1%, if any, ROR1 is phosphorylated on any tyrosine residues in the activation loop, confirming the *in vitro* data that ROR1 lacks robust kinase activity. These cellular phosphorylation experiments were performed under identical conditions to the TOPbrite reporter assays, providing a positive control of ROR1 functionality. While we did not observe autophosphorylation of ROR1 in response to Wnt5a stimulation, it is possible that another ligand for ROR1 exists which could activate the receptor in a different context.

It should also be noted that while we observed some residual tyrosine phosphorylation of ROR *in vitro*, it is possible that the protein preparations contained a small amount of active tyrosine kinase contaminant from the host cell lysate and we cannot rule out the possibility that wild type ROR1 and ROR2 completely lack catalytic activity. We were unable to isolate soluble cytoplasmic ROR protein containing classic catalytic inactivating mutations, as a negative control (Figure 1b, Mutations 1–4), however, our *in vitro* biochemical data indicate that the residual tyrosine phosphorylation of ROR is less than or approximately equal to that of the kinase-inactivating mutants of CAM-1 and MuSK (Figure 2).

Structures are available for several pseudokinase domains that, like ROR, contain non-consensus residues in the glycine rich loop, including Integrin-linked Kinase (ILK) [46], Vaccinia related kinase (VRK3) [47] and Rhotropy family members ROP2 [48] and ROP8 [49]. Of these pseudokinases, only ILK has been shown to bind ATP and none of these kinases has been shown to be enzymatically competent to phosphorylate any substrate or hydrolyze ATP. The greater than 10-fold average loss in activity of the P-loop mutants of CAM-1 and MuSK highlights the importance of the consensus P-loop in canonical kinase ATP binding and catalytic activity. However, restoration of the consensus P-loop in ROR does not result in an increase in enzymatic activity beyond that of the CAM-1 and MuSK mutant negative controls. This paradoxical phenomenon is not unique to ROR and has been reported for other pseudokinases, such as HER3 and STRADα [50], [51]. If these pseudokinases have evolved a predominantly non-catalytic role in signaling pathways, then this result is likely explained by an absence of selection pressure to retain other structural features, outside of the defined consensus motifs, that are essential for robust enzymatic activity.

As a pseudokinase, it is possible and perhaps likely that ROR may allosterically activate a functional kinase, as has been demonstrated with the pseudokinase STRADα and LKB1 [52]. As a kinase substrate, the C-terminus of ROR contains a proline-rich consensus sequence potentially capable of SH3 domain binding, as well as numerous tyrosines, which when phosphorylated, may be sites of SH2 domain-binding. Alternatively, the extracellular domain of ROR may serve as a node for protein-protein interactions; in which case, the kinase domain might participate through a substrate trapping mechanism.

In conclusion, we have shown that *C. elegans* CAM-1 is an active kinase *in vitro* while human ROR1 and ROR2 lack comparable catalytic activity in similar assays, suggesting an evolutionary divergence in at least some gene functions. Our data support the hypothesis that human ROR functions principally as an RTK-like pseudokinase. A more detailed study of these events will be essential to understand the physiological functions of ROR and may lead to the identification of the interaction targets of the kinase domain.

## Supporting Information

Figure S1 **ROR intracellular domains are pure and monomeric.** a) Coomassie stained SDS-PAGE gels of the ROR1, ROR2, ROR2 D482G and CAM-1 ICDs, 5 µg per lane. b) Comparison of the theoretical and experimental molecular weight of the wild type ROR1 and ROR2 ICDs as determined by MALLS.
(TIFF)

Figure S2 **Experimental replicate data of tyrosine autophosphorylation quantitation are consistent with** **Figure 2d** **.**
(TIFF)

Figure S3 **Validation of expression of ROR constructs in HEK293 cells.** a) HEK293 cells were transfected with various ROR constructs and FACS analysis performed using the anti-FLAG monoclonal antibody and PE-conjugated anti-mouse secondary. b) Lysates of HEK293 cells transfected with the various ROR constructs in Figure 4, and treated with combinations of Wnt3a and Wnt5a were tested for expression levels by immunoblotting using anti-FLAG monoclonal antibody.
(TIFF)

Table S1 **Observed phosphorylation sites in ROR1, ROR2 and CAM-1.** Phosphosite mapping was performed on the isolated ROR1, ROR2 and CAM-1 ICDs following incubation with magnesium and ATP. Tyrosine phosphorylation was observed in ROR1 and ROR2 only after phosphopeptide enrichment using TiO_2_ affinity chromatography, which was not needed for CAM-1. Phosphorylated residues are indicated in bold. *^a^*Bracketed sequences contain a phosphosite that could not be localized to a specific residue. *^b^*Peptides containing activation loop tyrosine residues.
(DOC)
