# Supplementary figures and images for: Evolutionary Divergence in the Catalytic Activity of the CAM-1, ROR1 and ROR2 Kinase Domains
Source: PLoS One. 2014 Jul 16;9(7):e102695. doi: 10.1371/journal.pone.0102695 (PMC4100928; doi:10.1371/journal.pone.0102695)

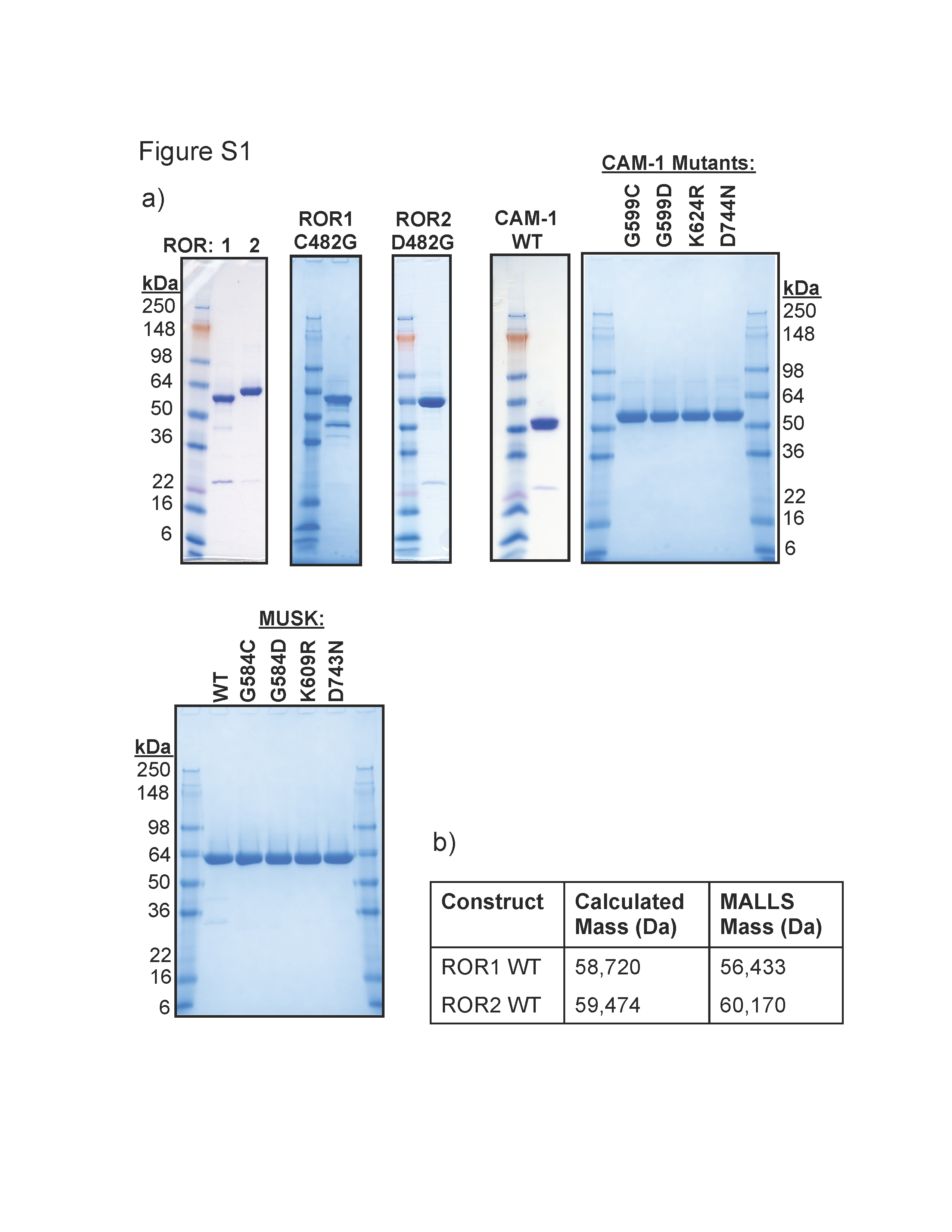

Supplement: Figure S1 — ROR intracellular domains are pure and monomeric. a) Coomassie stained SDS-PAGE gels of the ROR1, ROR2, ROR2 D482G and CAM-1 ICDs, 5 µg per lane. b) Comparison of the theoretical and experimental molecular weight of the wild type ROR1 and ROR2 ICDs as determined by MALLS. (TIFF) [file pone.0102695.s001.tiff]

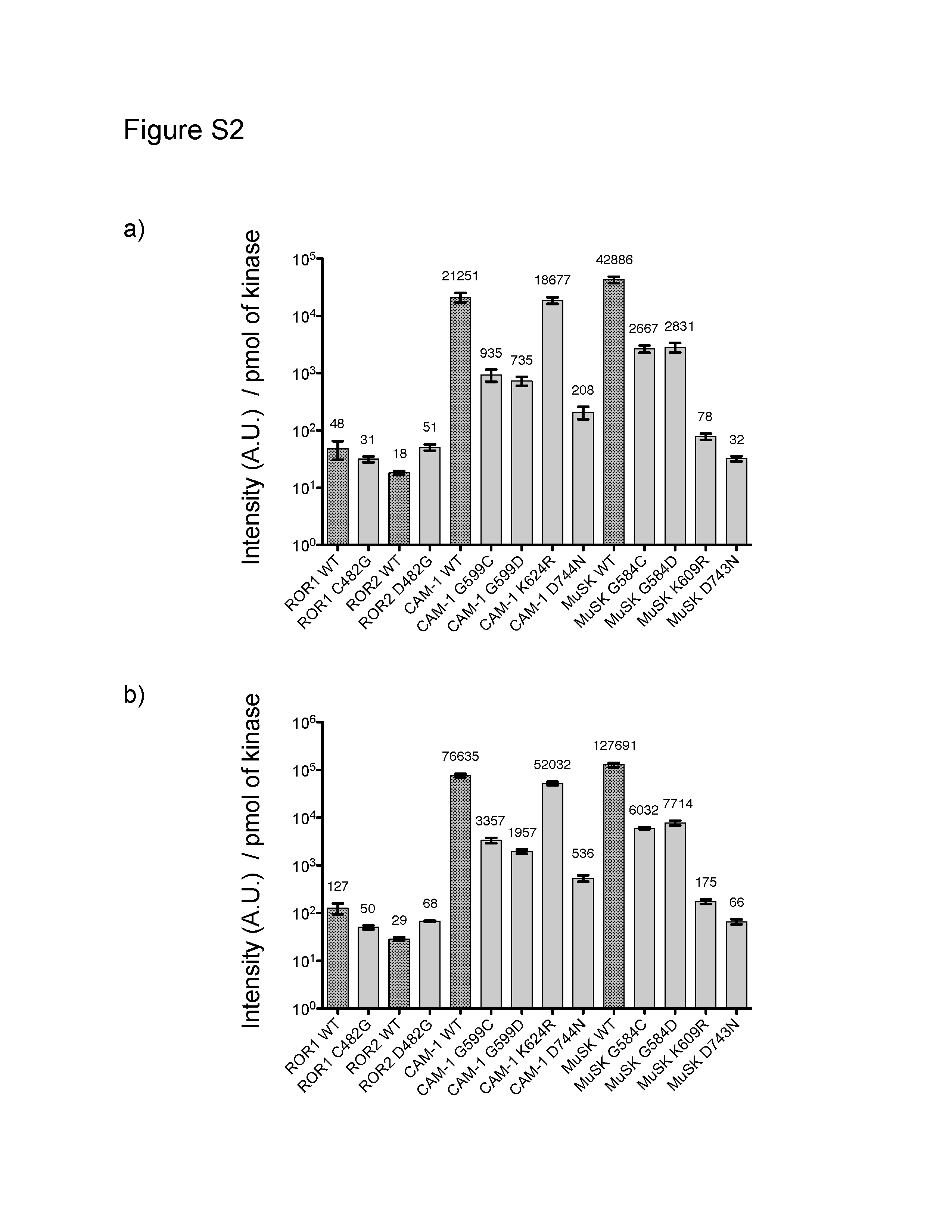

Supplement: Figure S2 — Experimental replicate data of tyrosine autophosphorylation quantitation are consistent with Figure 2d . (TIFF) [file pone.0102695.s002.tiff]

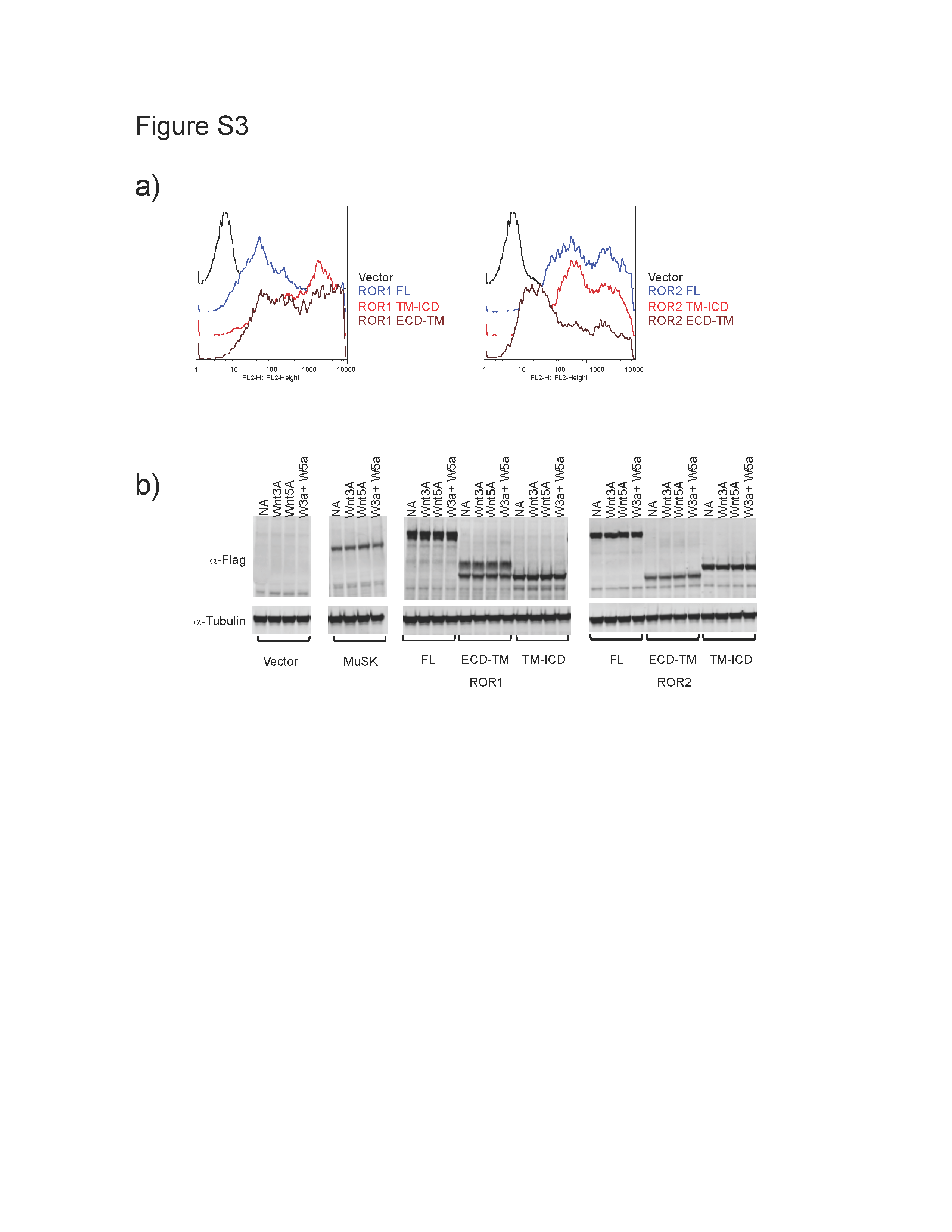

Supplement: Figure S3 — Validation of expression of ROR constructs in HEK293 cells. a) HEK293 cells were transfected with various ROR constructs and FACS analysis performed using the anti-FLAG monoclonal antibody and PE-conjugated anti-mouse secondary. b) Lysates of HEK293 cells transfected with the various ROR constructs in Figure 4, and treated with combinations of Wnt3a and Wnt5a were tested for expression levels by immunoblotting using anti-FLAG monoclonal antibody. (TIFF) [file pone.0102695.s003.tiff]
