# Supplementary material for: Evolutionary Divergence in the Catalytic Activity of the CAM-1, ROR1 and ROR2 Kinase Domains
Source: PLoS One. 2014 Jul 16;9(7):e102695. doi: 10.1371/journal.pone.0102695 (PMC4100928; doi:10.1371/journal.pone.0102695)
Supplement: Table S1 — Observed phosphorylation sites in ROR1, ROR2 and CAM-1. Phosphosite mapping was performed on the isolated ROR1, ROR2 and CAM-1 ICDs following incubation with magnesium and ATP. Tyrosine phosphorylation was observed in ROR1 and ROR2 only after phosphopeptide enrichment using TiO2 affinity chromatography, which was not needed for CAM-1. Phosphorylated residues are indicated in bold. aBracketed sequences contain a phosphosite that could not be localized to a specific residue. bPeptides containing activation loop tyrosine residues. (DOC) [file pone.0102695.s004.doc]

**TABLE S1.** Observed phosphorylation sites in ROR1, ROR2 and CAM-1.

| **ROR1 Peptide sequence** | **Number of PO^4-^** | **Residues** | **Calculated Peptide MWT** | **Observed ΔMWT ppm** | **Mascot Score** |
| --- | --- | --- | --- | --- | --- |
| [**SSS**]*^a^*APVQR | 1P | 434-441 | 910.39 | 2 | 53 |
| ELPL**S**AVR | 1P | 467-474 | 963.47 | 1 | 43 |
| **S**PH**S**DVGCSSDEDGTVK | 2P | 568-584 | 1935.65 | 2 | 45 |
| SPHSDVGC[**SS**] *^a^*DEDGTVK | 1P | 568-584 | 1855.69 | 2 | 51 |
| I[**S**] *^a^*DLGL**S**R | 1P | 631-638 | 939.44 | 6 | 33 |
| EI**Y**SAD**YY**R*^b^* | 1P | 639-647 | 1258.49 | 5 | 35 |
| SWEGLSSHTSSTTPSGGNATTQTTSLSA**S**PVSNLSNPR | 1P | 748-785 | 3883.74 | 10 | 53 |
| SSHTSST**T**P[**S**] *^a^*GGNATTQTTSL | 1P | 753-773 | 2101.88 | 6 | 59 |
| **ROR2 Peptide sequence** | **Number of PO^4-^** | **Residues** | **Calculated Peptide MWT** | **Observed ΔMWT ppm** | **Mascot Score** |
| ASA**ST**PQR | 1P | 434-441 | 896.37 | 3 | 57 |
| QLMA**S**P**S**QDMEMPLINQHK | 1P | 443-461 | 2292.99 | 1 | 39 |
| **S**PH**S**DVGSTDDDR | 2P | 569-581 | 1546.49 | -1 | 32 |
| EVYAADY**Y**K*^b^* | 1P | 639-647 | 1200.51 | 4 | 14 |
| AWGNLSNYNSSAQTSGASNTTQTSSLS**TS**PVSNVSNAR | 1P | 747-795 | 3925.73 | -1 | 60 |
| PSSHHSGSGSTSTGYVTTAPSN**T**SMADR | 1P | 859-886 | 2859.17 | 4 | 63 |
| **CAM-1 Peptide sequence** | **Number of PO^4-^** | **Residues** | **Calculated Peptide MWT** | **Observed ΔMWT ppm** | **Mascot Score** |
| HQNAHCSSAPSVINSAAN**S**A**YY**R | 2P | 475-497 | 2665.06 | -2 | 104 |
| LNGTS**T**PIMGR | 1P | 499-509 | 1226.56 | -1 | 46 |
| VPPHVEMTSLLPSAQHLGPPP**Y**PMDQHLQQAR | 1P | 510-541 | 3651.76 | -1 | 55 |
| FP**S**QEPIDDNSYK | 1P | 543-555 | 1619.66 | -2 | 71 |
| RFPSQEPIDDNS**Y**KVFEITPSQLSVR | 1P | 542-567 | 3132.5 | -1 | 61 |
| VFEITP**S**QL**S**VR | 2P | 556-567 | 1535.69 | -3 | 51 |
| VFEI**T**PSQLSVR | 1P | 556-567 | 1455.72 | -2 | 71 |
| IGEGQFGVVH[SGIY] *^a^*TSGLFAPEPMAVAVK | 1P | 570-598 | 3041.48 | -1 | 67 |
| TSYGSD**YY**K*^b^* | 1P | 724-732 | 1163.42 | -2 | 38 |
| **S**WMPVR | 1P | 737-742 | 855.35 | -2 | 27 |
| HLLECPHNCPTNI[YS] *^a^*LMVECWHENIER | 1P | 794-820 | 3531.51 | -1 | 42 |
| LQSW[SLAS] *^a^*PAH**S**ILQQHNNR | 1P | 831-850 | 2367.13 | -2 | 48 |
| AG**S**HSG**S**SGAGRPPTHQR | 1P | 851-868 | 1826.8 | -1 | 47 |
| VEGA**S**PLMK | 1P | 879-887 | 1011.45 | -2 | 55 |
